# Immersive virtual reality for pre‐registration computed tomography education of radiographers: A narrative review

**DOI:** 10.1002/jmrs.657

**Published:** 2023-01-19

**Authors:** Bridget Taylor, Glenda McLean, Jenny Sim

**Affiliations:** ^1^ Monash Imaging, Monash Health Berwick Victoria Australia; ^2^ Department of Medical Imaging and Radiation Sciences Monash University Clayton Victoria Australia

**Keywords:** Computed tomography, education, radiography, undergraduate, virtual reality

## Abstract

To be registered as a medical radiation practitioner, The Medical Radiation Practice Board of Australia (MRPBA) requires radiographers to be capable of performing computed tomography (CT) imaging examinations safely and effectively. Universities meet this requirement by offering practical CT training to radiography students on‐campus and during clinical placements. However, institutions face challenges when facilitating on‐campus CT practicum. Virtual reality (VR) has been suggested as a possible solution for radiography students to gain CT scanning experience. This narrative review explored relevant literature to investigate the potential for immersive VR to be incorporated into CT practicum. Benefits and limitations of this education technology are examined with resultant recommendations made for integration into the CT curriculum. Results found that VR enhances CT learning for students, increases confidence and raises motivation for the simulated CT task. CT simulation provides a viable alternative in the context of pandemic‐imposed restrictions and reduced CT placement duration. However, it remains debatable as to whether immersive VR truly enhances student learning compared with other VR modalities, such as computer‐based CT simulation. In addition, a lack of staff training, availability of resources and technical problems were flagged as limitations. We concluded that before immersive VR is integrated into CT education, significant optimisation of the simulation is needed. This includes ensuring VR scenarios are based on learning paradigms and feedback is integrated as part of simulation learning. Engaging clinical partners during the CT VR rollout is imperative to ensure successful transition of students from university learning to clinical placement.

## Graphical abstract

In this review, we sought to investigate the potential use of immersive virtual reality (VR) for computed tomography (CT) simulations during pre‐registration radiographer training at a tertiary level. We found that immersive VR simulations performed at university are an accessible mode of CT simulation that can promote trial‐and‐error style learning and progress student confidence surrounding CT scanning. This is significant as this mode of simulation has the potential to improve student clinical reasoning and foster critical thinking amongst CT students before they enter clinical placement.

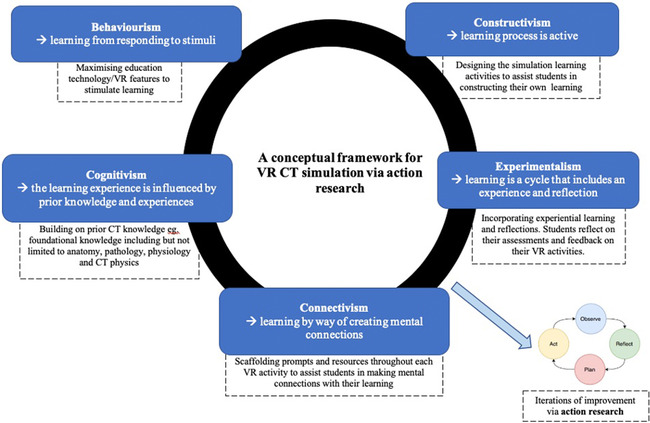

## Introduction

The current registration requirements under the Medical Radiation Practice Board of Australia (MRPBA) professional capabilities for medical radiation practitioners mandate that radiographers are able to perform computed tomography (CT) imaging in a safe and effective manner in accordance with departmental CT protocols.^1^ This registration requirement is met by tertiary institutions through integrating CT knowledge and practical simulations into their radiography curricula.^2^ However, universities face a number of challenges to achieving practical simulations in‐person, including the immense cost of an onsite CT scanner. In more recent times, the problem of adequately preparing students for CT was further exacerbated with the COVID‐19 pandemic reducing practical laboratory time at university and clinical placement. With limited exposure to practical CT experience, students may be underprepared for their CT placement. Hence, innovation in CT education strategies is needed to ensure this key capability continues to be met.

Virtual reality (VR) simulation has been posed as a solution for healthcare students to gain experience performing clinical tasks in a virtual environment.^3^ Although there is no standardised definition for VR, it describes the experience of being immersed inside a 3D environment through digital simulation.^4^, ^5^ There are different types of VR that vary in terms of immersivity, where non‐immersive VR gives interaction with a 3D display on a computer monitor and semi‐immersive VR allows fusion of digital information with the real world. Fully immersive VR systems comprise of hardware and software components, including sophisticated head‐mounted displays (HMD), hand controllers, motion trackers and the user interface.^6^ Some newer VR systems available have inbuilt motion tracking in the HMD. The user hence experiences full immersion into a 3D environment.^4^, ^6^ Currently, immersive VR simulations have been used in nursing, medicine and dental curricula as a way of consolidating clinical decision making and exposing students to challenging clinical scenarios without being in a dedicated clinical setting.^7^, ^8^, ^9^ Non‐immersive VR approaches to CT simulations are currently available in tertiary medical imaging (MI) faculties, such as the computer‐based Siemens CT simulation and NETRAD remote access CT simulations, which offer students a similar experience to physically operating a CT scanner.^2^, ^10^, ^11^ This includes setting scan parameters and creating multi‐planar reformats.^2^, ^10^, ^11^

Although there are studies that explore student perspectives on immersive VR in other pre‐registration healthcare settings, few fully immersive VR systems have advanced beyond developmental stages to be fully integrated into tertiary healthcare education.^12^ This includes integration into pre‐registration CT education, where the literature is even more limited.^11^

This review aims to explore enablers and barriers to implementing immersive VR simulation into the pre‐registration radiography CT curriculum by performing a narrative review of the literature surrounding the use of VR in undergraduate MI and other allied health faculties. Current approaches to integrating VR into the undergraduate curriculum of other allied health faculties will be first discussed before exploring if the same strategies can be applied to implement immersive VR into pre‐registration CT simulation and emulate a holistic clinical scenario.

## Method

The following electronic databases were searched for relevant literature: PubMed, Elsevier Science Direct, Monash University Library database. Search terms which were used for each database are displayed in Table 1.

**Table 1:** Keyword search strategies used for each database in the literature search.

Database	Search Strategy
PubMed	*Free Text*: (computed tomography) AND (virtual reality OR immersive virtual reality) AND (healthcare) AND (education)
Scopus Science Direct	*Free Text*: (computed tomography) AND (virtual reality OR immersive virtual reality) AND (healthcare) AND (education)
Monash University Library	*Free Text*: (computed tomography) AND (virtual reality OR immersive virtual reality) AND (healthcare) AND (education)

Review articles, meta‐analyses, randomised controlled trials and pilot studies that were published from 2015 to 2022 were included in the search strategy.

**Figure 1:**
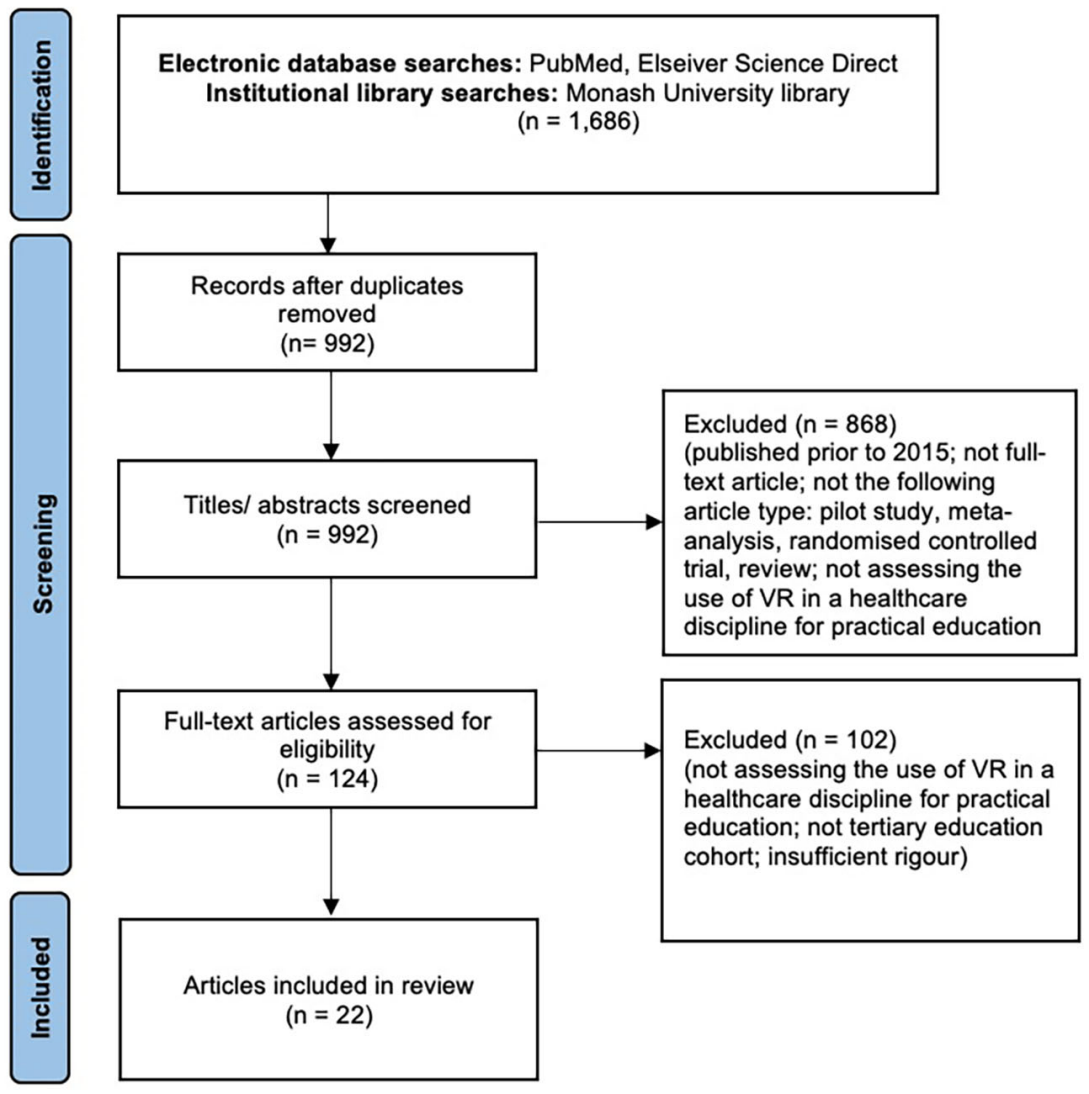
PRISMA flow diagram for the literature search method used in this review.

Articles were appraised manually based on the relevance of their title, key words and abstract to our research question. Articles with perceived applicability to the research question were included and the full text reviewed. A flowchart of our method can be seen in Figure 1.

## Results

Table 2 summarises the 22 articles used in the review.

**Table 2:** Summary of 22 articles included in the review.

Author	Study type and aim	Participants	Method	Key findings
Adhikari et al. 2021^13^	Mixed‐methods feasibility study Investigate impact of VR sepsis game on nursing student self‐efficacy and perception of its use in the curriculum	19 nursing students Single tertiary institution	Students accessed VR sepsis game Self‐efficacy measured using NASC‐CDM© scale pre‐ and post‐intervention Informal, semi‐structured interviews	Confidence significantly increased and anxiety reduced (*P* < 0.001)
Dahri et al. 2019^23^	Qualitative evaluation study Assess how pharmacy students perceive VP sim Investigate how to integrate VR into curriculum	180 pharmacy students surveyed 6 pharmacy students interviewed	4 VP sim's over 3 years Survey and focus‐group interviews post‐intervention	Strongly agreed VP was a valuable learning experience Experience was engaging
De Ponti et al. 2020^15^	Qualitative evaluation study Investigate how medical students perceived computer VR sim for patient interactions	115 6th year medical students Single tertiary institution	21 VR simulations over 3 months 4‐point Likert scale survey post‐intervention	84% perceived VR platform to be suitable for training bedside interactions 28% felt technical problems were an accessibility barrier
Fealy et al. 2019^9^	Scoping review Investigate implementation of VR into nursing curricula Assess the need for more research in this area	*n* = 2 articles	Scoping review	Collaboration with IT and nursing during software development is important VR is able to immerse students into high‐risk scenarios
Gregory et al. 2015^22^	Systematic review and qualitative study Investigate why VR simulations have not been adopted widely in tertiary education by educators	223 educators 134 tertiary institutions 28 countries	Survey questionnaire with 17 ‘pre‐designed’ answers or could give free‐text response	54% of educators currently using VR believe their institution does not provide technical support 36% of educators currently using VR will use it in the future
Gunn et al. 2018^17^	Mixed methods study Investigate how VR learning affects technical skill development in radiography students	45 first year radiography students single tertiary institution	two groups: computer VR sim for AP foot vs roleplay in x‐ray laboratory for PA scaphoid projection assessor used rubric to evaluate student technical performance	x‐ray skills improved by 4.75% when using VR, slightly better than role‐play
Gunn et al. 2021^11^	Qualitative evaluation study Investigate student perception of VR for CT learning in MI and RT students	28 MI students 38 RT students single tertiary institution	5‐point Likert scale survey and statistical analysis regarding MI student experience using VR for CT sim	54% had maximum engagement, and had confidence score 1.02 (mean) higher than those not engaged Educators unable to answer student questions about the VR
Joda et al. 2019^8^	Systematic review Investigate new developments of AR/VR relating to dental medicine	*n* = 16 articles	Systematic review with modified PICO framework	Use in training motor skills and practicing surgeries 3D VR a better learning experience than 2D Cost/benefit evaluation necessary
Kardong‐Edgren et al. 2019^5^	Literature review Review definitions of VR to recommend a more precise way for definition	*n* = 13 articles		Recommend that authors define VR/AR when publishing Include levels of immersion in definition
Kavanagh et al. 2017^18^	Systematic review Investigate problems preventing wider integration of VR into education	*n* = 99 articles	systematic review and thematic analysis	VR motivates students to learn through gamification, constructivism and collaboration Problems: cost, training, technical problems, usability
King et al. 2018^25^	Literature review Investigate how VR has evolved in healthcare education	*n* = 8		VR enhances learning opportunities in clinical learning
Lee & McInerney 2022^16^	Mixed methods study Investigate computer‐based VR CT sim for pre‐placement preparation	60 third year Radiography students Single tertiary institution	students engage with VR CT sim for 5 hours total Likert survey and free‐text response questions	34% suggested VR sim platform was difficult to use Suggested the ability to learn through making mistakes enhanced CT learning experience
Lee et al. 2020^2^	Mixed methods study Investigate whether students accessing computer CT sim impacted knowledge before and after clinical placement	62 third year Radiography students Single tertiary institution	Participants split into CT sim group and physical scanner group 1.5 hours access each Knowledge tested before and after placement in form of test Likert survey about experiences	Improved knowledge about CT parameter selection after using CT sim software (*P* < 0.001) No difference in test results between in‐person and virtual simulation (*P* = 0.6)
Liaw et al. 2021^3^	Qualitative evaluation study Investigate how students and educators experienced immersive VR learning for interprofessional practice Suggest method for optimising integration into curriculum	30 students from 6 healthcare disciplines, final 2 years of study 12 facilitators Single tertiary institution	Split into groups of 6 students and 2 facilitators, simulating interprofessional care situations using the VR Focus group and single person interviews following intervention	Perceived as a way to collaborate in a ‘safe’ virtual environment Technological fascination was engaging for students Technical issues were a barrier
Liley et al. 2020^10^	Pilot study Investigate student confidence and attitudes towards remote CT sim	Third year radiography students, 21 pre‐ & 23 post‐placement Single tertiary institution	Students used remote access CT sim in 2 hours sessions Pre‐ and post‐placement 5‐point Likert surveys	Students felt that remote CT sim did not prepare them for placement Highlighted convenience & repeatability
López Chávez, Rodríguez & Gutierrez‐Garcia 2020^24^	Mixed methods study Investigate differences in VR teaching environments and impact on medical student learning	78 undergraduate medical students Single tertiary institution	4 groups given clinical case in different learning environments: 2D, 2D gamified, 3D and immersive VR Students took written examination following simulation Surveyed using 12‐point Likert questionnaire	Students perceived gamification as beneficial for learning, scored higher in 3D and 2D gamified simulations Information retention affected by simulation design approach
O'Conor et al. 2021^7^	Pilot study Determine whether immersive VR is beneficial to student confidence and skill level Find improvement areas for software and integration into curriculum	105 first year radiography students	Students guided through learning module with VR headset and hand controllers Simulated positioning and exposing a patient in x‐ray suite Online survey after	58% enjoyed the simulation 58% wanted more time to feel confident using the VR Self‐reported to have improved x‐ray parameter selection
Radianti et al. 2020^12^	Systematic review Review how budget and expensive VR systems are designed for higher education use	*n* = 38 articles	systematic review with thematic analysis coding matrix	Most VR systems are in experimental development Few systems based on learning theories
Saab et al. 2021^14^	Qualitative evaluation study Investigate nursing student perceptions of integrating VR into their curriculum	26 nursing students Single tertiary institution	Students engaged with VR sim Individual and focus group interviews conducted regarding their experiences Thematic analysis	VR is engaging and practical Helps build confidence in clinical skills when adjunct to traditional learning methods Lack of feedback and resources perceived as a barrier
Sapkaroski, Mundy & Dimmock 2020^19^	Split‐cohort study Investigate MI student perspectives of immersive VR vs in‐person roleplay	76 first year MI students Single tertiary institution	2 groups: group 1 used immersive VR to perform hand x‐ray, group 2 used roleplay 5‐point Likert scale survey and open‐ended questions post‐intervention with thematic analysis	Roleplay group expressed desire for more time VR group expressed positivity with working through task in their own time
Uppot et al. 2019^20^	Narrative review article Investigate how imaging departments have integrated VR and AR	3 imaging departments	Narrative review	Used immersive VR to simulate responding contrast anaphylaxis reactions Raised ergonomic issues (e.g. neck pain and cybersickness)
Uruthiralingam & Rea 2020^21^	Systematic review Investigate best practice for use of VR and AR in anatomy education	*n* = 31 articles	Systematic review	VR widely supported for education applications Raised student motivation to study 3D VR gave increased spatial understanding of anatomy

Abbreviations: AR = augmented reality; CT sim = computed tomography simulation; IT = information technology; MI = medical imaging; NASC‐CDM© = nursing anxiety and self‐confidence with clinical decision making; RT = radiation therapy; VP sim = virtual patient simulation; VR sim = virtual reality simulation; VR = virtual reality.

## Discussion

### Benefits and enablers of VR

#### Student confidence

VR simulations relating to clinical tasks have the potential to enhance student confidence performing these tasks when entering the real‐life clinical environment.^13^, ^14^, ^15^, ^16^ One study in which radiography students were surveyed about their learning experience following the use of weekly VR x‐ray examination simulations found that students perceived that the VR had improved their confidence and skill development with setting x‐ray technical factors, such as kVp, mAs and source‐to‐image distance (SID).^7^ A similar 2018 study confirmed that radiography students were able to improve their general x‐ray technical skills through VR simulation at a level that was similar to or slightly better than those gained in traditional skills laboratory sessions.^17^ This illustrates that VR simulations aid MI students to feel self‐assured with their improved radiographic skills following the simulation activities. The same benefits of improved CT capability may be considered for VR CT as it is technically similar to other radiography tasks.

As VR removes students from the physical clinical environment, students are able to practise in high‐risk, authentic clinical scenarios and make mistakes without jeopardising patient safety or negatively impacting department workflow and resources.^9^, ^16^, ^18^ Indeed, MI students perceived immersive VR to be accessible and beneficial in trial‐and‐error style learning.^19^ There is limited literature specifically surrounding the impact of immersive VR simulations on student confidence in CT. Two studies found that the opportunity to practise CT skills on a computer‐based VR platform helped MI students feel more prepared for real‐life scanning given the risks and high radiation doses involved with CT examinations.^11^, ^16^ Students may also be exposed to adverse events, such as contrast reactions through simulation.^20^ This may reduce anxiety about the confronting nature of anaphylaxis.^20^ This theme is common in pre‐registration nursing education, where VR simulations of clinical scenarios have been shown to alleviate student anxiety surrounding high stakes clinical incidents as it provides a virtual environment where both patient and student remain safe.^3^, ^13^

Clearly, VR has a place in better preparing students to be safe and confident in their expected roles during clinical placement. Confidence, however, was self‐reported in all of the studies referenced above as surveys were used in their methodology. As their results are based on self‐reported confidence, which is inherently subjective, findings of these articles may also be subjective. Furthermore, improving student confidence may not translate to them gaining clinical competence. However, VR that increases confidence and skills allows students to be more willing to practise their CT scanning during clinical placement and go beyond their comfort zone to acquire new knowledge and skills.

#### Motivation and engagement

Virtual reality simulations engage healthcare students and motivate them to participate and learn during practical simulations.^14^, ^18^, ^21^ Simulation encourages students to take an active role in their own learning compared with traditional clinical experiences, where time and hierarchical pressures may limit their engagement.^3^ This may be a relevant motivator for CT students as VR offers a safe learning environment to practise scanning in their own time and in a non‐judgmental space. Nursing students who participated in a VR clinical simulation were more engaged and were therefore more likely to remember the skills taught during the simulation.^14^ However, the major downfall of studies exploring student motivation with VR through interviews and surveys is that a student's perception of what is ‘engaging’ is subjective and individual. The belief that VR is a motivating technology may not necessarily hold true across other healthcare disciplines, including CT education.

Virtual reality fascination is a common theme in the literature, with suggestion that VR simulations which are gamified result in a higher student engagement as they find enjoyment in the technology.^18^ VR, however, is a new technology used in healthcare education as illustrated by the articles included in this review dating from 2015 to 2022. Consequently, the increased motivation and engagement that VR affords may be the result of novelty. Student engagement with the technology may hence diminish over time as novelty fades.^18^

#### Flexible learning

Unlike simulations performed on physical CT scanners (i.e. in‐person simulation), VR simulations offer flexible learning options for students and facilitators alike. The clear benefit of VR CT simulation is that a CT scanner is not required.^11^ In the context of MI, students are also not permitted to take a radiation exposure without the presence of a licensed supervisor.^7^ With the use of immersive VR, MI students who are unable to access a CT scanner with licensed staff during their on‐campus training may engage in a simulated CT experience. The use of remote‐access NETRAD CT simulation has been studied as a computer‐based simulation which involves students making bookings to use a CT scanner remotely via their computer device.^2^, ^10^ The ability to perform the simulation independently 24/7, as well as the option to repeat the simulation activities, promotes learning and strengthens clinical reasoning for students.^2^, ^10^ This is relevant as large caseloads experienced during clinical CT placements may limit opportunities for students to understand the critical learning unfolding before them.

The idea that students may access VR at an appropriate time and place may not be transferrable to immersive VR applications. This is because the hardware aspects of VR limit the setting and number of students that may access the simulation. Unlike a computer‐based VR which may be accessed on individual devices at any given time, depending on how the institution chooses to distribute VR devices, VR equipment may need to be individually rented by students or a dedicated space with VR hardware setup specifically for use by the whole MI faculty. This limitation is much smaller compared with the timetabling and licensed staffing constraints of performing simulations on a CT scanner.

#### Usefulness for CT learning

Before deciding to integrate immersive VR into CT education, the question must be asked whether or not immersive VR is useful in enhancing student learning and transferring into clinical practice. Ultimately, the usefulness of VR rests with its ability to effectively meet learning outcomes set by CT educators. However, this is divisive among the literature.^21^ Two studies which compared VR simulations to in‐person roleplay for radiography practical sessions rated that both methods have a similar effectiveness in enhancing student technical competence.^17^, ^19^ Students perceived that VR simulations enhanced their clinical reasoning and according to x‐ray parameter selection at a similar level to in‐person simulations.^17^, ^19^ Conversely, one study revealed that student knowledge about CT‐related problem‐solving, image quality and protocol selection improved from before using computer‐based VR to after as there was a statistically significant difference in test scores (*P* < 0.001).^2^ However, there was no statistically significant difference in test results between students using VR and those operating a physical CT scanner (*P* = 0.6).^2^ Overall, this shows that students may be able to improve their CT technical knowledge and decision making through VR simulations and in‐person simulations. Hence, both education strategies may be equally useful regarding CT student learning depending on the resources available in the education setting.

### Limitations and barriers of VR

#### Cost and resources

The cost of tertiary VR solutions described in the literature varies. Some studies identify that significant overhead costs associated with purchasing hardware, developing software and training staff have prevented implementation of VR simulations in many university faculties.^18^, ^22^ A lack of funding, including cost of initial purchasing and ongoing VR maintenance, has been shown to significantly hinder the implementation of virtual worlds across all faculties.^18^, ^22^

Conversely, other studies have suggested that VR simulations are more cost‐effective when compared to facilitating a true clinical simulation.^2^, ^9^, ^21^ This is relevant to the discussion for CT as this area of radiographer training is extremely resource intensive. It is clear that a cost–benefit evaluation needs to be performed to justify whether the benefits of VR CT simulation outweighs its exorbitant cost. While at the time of this writing, the impact of COVID‐19 on placement has subsided, it is an accepted reality that COVID‐19 will not be our last pandemic. Hence, it is prudent when planning for pre‐clinical preparation to consider VR as a sustainable education solution. While the cost may be prohibitive, a pandemic can result in significant loss of placement time due to the need to limit resourcing and restrict the number of personnel permitted in a small space.

#### Technical problems

Software faults are the most common problem faced by VR facilitators and users when using the technology for education.^18^ Some of these faults reported in the literature include problems with remote access, internet connection, microphone and lag.^3^, ^10^, ^16^ For instance, one VR simulation for medical student training found that connection problems affected one third of the participating cohort.^15^ Lag and bugs during immersive and non‐immersive VR simulations have also been reported by MI students as a barrier to full engagement with the simulations.^10^, ^16^ It could be hypothesised that technical problems are prevalent in VR simulations due to the infancy of the technology in tertiary education applications.

#### Lack of resources and training

A lack of training for educators using VR systems may result in the technology not being used to its full potential during simulations in CT education. Educators report being unsupportive of the use of VR in tertiary education due to perceived difficulty of use and lack of training from their institution in becoming proficient to teach with VR.^22^ Initial staff inductions to the technology also present a significant overhead cost as trainers and teaching staff are required to be paid for their time.^18^ This may create a financial barrier for some institutions, resulting in a lack of meaningful support. Educators insufficiently trained to use VR may be unable to answer students' technical questions about the simulation, which may in turn limit student engagement with the learning experience.^11^

#### Lack of human interaction

By nature of being in a virtual world, there is suggestion that VR clinical simulations have limited human interaction, extending to the lack of opportunity for educators to give feedback to students. Unlike in‐person clinical scenario simulations, it has been noted that VR limits the ability for healthcare students to learn soft skills as there is no simulated patient interaction.^10^, ^13^ The focus of some simulations may rest more on technical skill development and not on learning to deliver patient care, which again is a key capability of registered radiographers as stated by the MRPBA.^1^ However, there are VR simulations that are designed to enhance emotional intelligence and soft skills. When a computer‐based VR simulating patient/medical student interactions was used, 84% of surveyed medical students believed that the VR had enhanced their bedside manner, highlighting that VR can develop soft skills.^15^ Hence, a lack of human interaction in the context of CT education is only a limitation if the VR environment is designed to focus solely on technical skills.

A common theme in the literature is that healthcare students believe that VR simulations do not currently offer the ability to receive immediate feedback from supervisors, and this consequently hinders their learning. Students who accessed a CT scanner remotely through a computer‐based VR set‐up had difficulty engaging with the simulation as there was no facilitator involved.^5^, ^8^, ^14^ Similarly, it has been found that radiography students using VR simulations for general radiography practical sessions believe that a lack of immediate feedback from supervisors regarding their radiographic technique hindered their skill development.^5^ In both of these studies, feedback was not integrated into the VR simulation. Consequently, a lack of supervisor feedback will be a limitation for VR in CT education if the simulation fails to include supervisor feedback.

#### Effectiveness of immersive VR

Another question that should be raised is whether immersive VR is more effective than non‐immersive VR for CT simulation. It has been suggested that immersive VR is useful as it allows students to engage with clinical tasks with realism and experimental interaction that enhances contextual learning.^3^ Alternatively, many studies included in this review examined the use of non‐immersive VR technology, such as computer‐based simulations accessed on student computer devices, which was found to have enhanced student learning.^10^, ^15^, ^23^ One study investigated the examination performances of medical students after engaging with the MediTool clinical simulation in computer‐based 2D, gamified 2D, 3D and immersive VR environments.^24^ The median test score was highest for students using gamified 2D MediTool, with immersive VR only ranking 3rd.^24^ This result, although not found to be statistically significant (*P* = 0.4036), highlights that non‐immersive VR technologies may be equipped to answer the learning outcomes for a simulation. Indeed, there appears to be a small trend for non‐immersive computer‐based VR simulations to be used for CT education and radiation therapy, where students access the simulation on individual devices in and/or out of class.^2^, ^10^, ^11^, ^16^ In the context of CT education, introducing immersive VR may be excessive when a computer‐based simulation can aid students in gaining essential clinical reasoning.

It is acknowledged that the setting in which students access immersive VR simulations may also influence feasibility factors such as cost, training effectiveness and quality of the CT learning experience. Many studies did not clearly define the exact settings in which the students accessed VR simulations, including descriptions of where and with whom exactly the simulation was accessed. It is hence difficult to explore whether setting impacts feasibility factors but is suggested for future research endeavours.

### Integrating Immersive VR into CT education

There are a number of important considerations that need to be addressed in order for VR to be successfully integrated into CT education. Firstly, it is recommended that more research be conducted to further optimise the way in which immersive VR could be used for CT simulations in a pre‐registration CT curriculum as there is currently very limited literature published on this subject.

The recommendations listed below will focus on key areas where the immersive VR could be optimised to enhance the simulated learning experience. They are centred around the benefits and correcting for the limitations of immersive VR simulations as discussed above. Although some articles used in this review surround VR in other healthcare faculties, these key areas have been chosen as they are able to be transferred to pre‐registration CT education.

#### Design of the immersive VR simulation

Considerations for designing immersive VR simulations were not widely discussed in the articles included in this review. Most articles relating to healthcare education created VR simulations which emulate clinical scenarios that would have otherwise been simulated in a clinical skills laboratory or on clinical placement.

Using prototypes of the simulation, immersive VR environments may be created in collaboration with software developers and educators to best meet the learning objectives for the simulation.^4^, ^25^ In practice, however, this may not be the case as many articles included in the review did not describe the process of how their VR simulation was created. Consequently, the idea that the development of a simulation as a collaborative venture between vendor and tertiary institution may not be a common occurrence. This lack of partnership may result in VR simulations not being cemented on learning paradigms when achieving realism and interactivity are instead a higher priority.^12^

1. *Base the simulation on education theories and learning paradigms*

**Figure 2:**
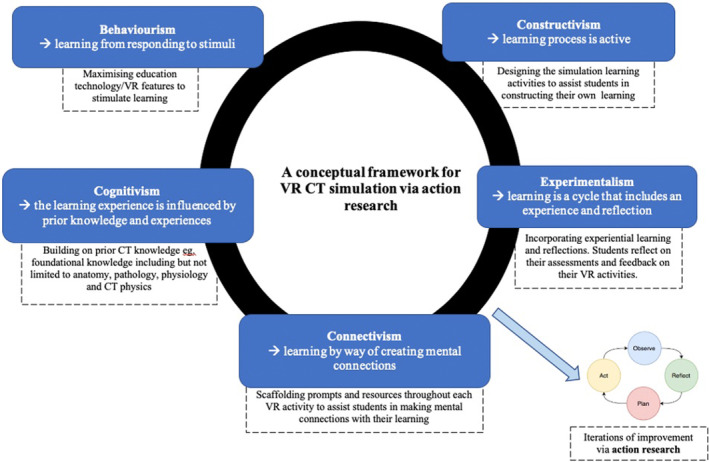
An illustration of how the education learning paradigms, as proposed by Radianti and Majchrzak,^12^ can be adapted as an conceptual framework for VR CT simulation learning. The VR CT simulation activities are improved with each iteration of cyclical action research.

VR simulations for CT that are not based on learning theories and paradigms may fail to maximise the simulation technology to improve student understanding and better prepare them for clinical practice. There is hence a need to design or adopt an education framework for the VR CT simulation learning. Figure 2 is an example of how learning paradigms can be adapted as a framework for immersive VR CT simulation.^12^ This will ensure that radiography students are able to acquire CT knowledge, skills and confidence as part of their registration requirement. Communication and other soft skills may be integrated into the VR simulation if these are designated as part of the learning outcomes.

2 *Embed assessment as part of the simulation*

There are a number of ways that students can engage with VR simulations described in the literature. The possibility to integrate immersive VR simulations into assessments has also been flagged. Two studies examined in this review did not exclusively use VR as a platform to undertake assessments on, but instead tested content relating to the simulations in a written assessment.^2^, ^24^ For VR learning to be authentic, it is imperative that assessment of student learning should likewise be conducted in the VR environment, thereby emulating clinical workplace. However, one study revealed that radiography students were opposed to the idea of VR being used for major assessments, such as clinical skills examinations, but were in favour for its use in low stakes assessments.^7^ This is indicative of the fact that VR has not been accepted as an inherent part of mainstream learning and highlights the importance of incorporating assessments as part of VR simulation to ensure learning outcomes are met. In addition, instant supervisor feedback should also be integrated into the simulation where students can reflect on their learning and performances during the CT simulation as providing immediate feedback has been highlighted as an important design feature.^19^

3 *Improve simulation learning* via *action research*

As part of the collaboration between staff, students and software developers, the simulation learning should be improved via the iterative cyclical process of action research. This ensures technical problems and bugs are removed, learning outcomes are constantly refined and met as well as improving the spatial realism of the simulation so that the simulated CT learning and experiences are comparable to a physical scanner.

Further, artificial intelligence (AI) and machine learning could improve realism and enhance learning in VR simulations. AI and VR simulations have been used in conjunction for medical training, where AI feedbacks the consequences of the user's actions.^26^ In the context of CT simulation, AI could be used in biofeedback devices such as heart rate monitors and pulse oximeters, as well as in creating a ‘virtual patient’ that elicits physical responses to the user's scanning technique. For example, heart rate increases on the monitor when the patient is simulated to have an allergic reaction. Through this, students may better engage in trial‐and‐error style learning with a greater understanding for the consequences of their scanning technique. Although AI was not discussed in the articles included in this review, potentially because it is not yet a mainstream feature, it is acknowledged as an avenue for future action research in order to improve simulated learning.

#### Sustainability: Set aside resources for training and induction

Sufficient resources must be set aside for continuous delivery of VR simulations. The methods discussed in the literature for training staff using VR in healthcare education is limited. Staff engagement is critical to successful VR delivery and ensuring that staff are comfortable with using the VR should be a continued priority. It is recommended that there should be a focus on staff training to avoid situations where the educator is unable to guide students through CT simulations. Training may be accessed through liaising with vendors who supply trainers to educate staff through in‐person sessions.^4^ Online video tutorials may also be a valuable learning tool to help staff gain competence with the VR software and hardware with the added bonus of being repeatedly accessible to staff at a convenient time.

Once educators gain familiarity with the equipment, in‐person tutorial sessions for students take place to facilitate the transfer of knowledge from educator to student and for the student to familiarise themselves with using the equipment prior to undertaking the simulated task.^2^, ^7^ Consensus among articles relating to VR in health care is to perform 0.5–2 hours VR sessions for small groups at regular time intervals.^7^, ^15^, ^21^, ^23^ Delivering the simulations at regular time intervals is critical to maintaining the knowledge students acquire during simulations. Videos instructing students how to use the software and hardware are used to deliver training to students.^7^ Despite this, a portion of healthcare students have expressed the need for an increased amount of time familiarising themselves with the VR before commencing the actual learning from the simulation.^7^, ^21^ If students have a negative experience learning how to use immersive VR, they are unlikely to embrace the benefits of VR learning. This idea, combined with the fact that no studies included in this review specifically evaluated how successful student training methods were, suggests that more attention needs to be directed to ensuring that students are adequately trained to use the immersive VR software.

#### Engage clinical partners in the VR CT simulation planning and implementation

It is also important to engage with the clinical partners of the university to gain their support for the use of immersive VR for CT education. Engaging them as part of the VR CT simulation planning and rollout is integral to winning their support as students transition from university learning into clinical placement. Such dialogues between the university and the clinical providers will ensure a seamless transition of student learning in this current era of constant technological changes.

## Conclusion

Immersive VR in CT simulation shows not only benefits but also considerable limitations that need improvement to be successfully integrated into the pre‐registration CT curriculum. The main enablers of the technology include its positive effect on student confidence, motivation and CT learning and offering flexible learning. Conversely, the main limitations of the technology include technical problems, cost, lack of training and reduced human interaction. In order to integrate immersive VR into CT education, the simulation needs to be designed based on learning paradigms with integrated feedback and assessments, plus a focus on staff training. Furthermore, additional research needs to be conducted to compare whether immersive VR is beneficial over non‐immersive VR for CT simulations, such as computer‐based CT simulation. Last but not least, inviting clinical partners to engage with universities and students during planning and rollout is imperative to embrace education technology that improves the quality of the graduating CT workforce against the constant threat of pandemic‐imposed restrictions and reduced CT placement opportunities.

## Conflict of Interest

The authors declare no conflict of interest.
